# Proton Therapy for Prostate Cancer: Challenges and Opportunities

**DOI:** 10.3390/cancers14040925

**Published:** 2022-02-13

**Authors:** Darren M. C. Poon, Stephen Wu, Leon Ho, Kin Yin Cheung, Ben Yu

**Affiliations:** 1 Comprehensive Oncology Centre, Hong Kong Sanatorium & Hospital, Hong Kong 999077, China; mc_poon@clo.cuhk.edu.hk; 2 Medical Physics Department, Hong Kong Sanatorium & Hospital, Hong Kong 999077, China; leon.cc.ho@hksh.com (L.H.); kycheung@hksh.com (K.Y.C.); benyu@hksh.com (B.Y.)

**Keywords:** prostate, proton therapy, magnetic resonance imaging

## Abstract

**Simple Summary:**

Reported clinical outcomes of proton therapy (PT) for localized prostate cancer are similar to photon-based external beam radiotherapy. Apparently, the dosimetric advantages of PT have yet to be translated to clinical benefits. The suboptimal clinical outcomes of PT might be attributable to inadequate dose prescription, as indicated by the ASCENDE-RT trial. Moreover, uncertainties involved in the treatment planning and delivery processes, as well as technological limitations in PT treatment systems, may lead to discrepancies between planned doses and actual doses delivered to patients. In this article, we reviewed the current status of PT for prostate cancer and discussed different clinical implementations that could potentially improve the clinical outcome of PT for prostate cancer. Various technological advancements under which uncertainties in dose calculations can be minimized, including MRI-guided PT, dual-energy photon-counting CT and high-resolution Monte Carlo-based treatment planning systems, are highlighted.

**Abstract:**

The dosimetric advantages of proton therapy (PT) treatment plans are demonstrably superior to photon-based external beam radiotherapy (EBRT) for localized prostate cancer, but the reported clinical outcomes are similar. This may be due to inadequate dose prescription, especially in high-risk disease, as indicated by the ASCENDE-RT trial. Alternatively, the lack of clinical benefits with PT may be attributable to improper dose delivery, mainly due to geometric and dosimetric uncertainties during treatment planning, as well as delivery procedures that compromise the dose conformity of treatments. Advanced high-precision PT technologies, and treatment planning and beam delivery techniques are being developed to address these uncertainties. For instance, external magnetic resonance imaging (MRI)-guided patient setup rooms are being developed to improve the accuracy of patient positioning for treatment. In-room MRI-guided patient positioning systems are also being investigated to improve the geometric accuracy of PT. Soon, high-dose rate beam delivery systems will shorten beam delivery time to within one breath hold, minimizing the effects of organ motion and patient movements. Dual-energy photon-counting computed tomography and high-resolution Monte Carlo-based treatment planning systems are available to minimize uncertainties in dose planning calculations. Advanced in-room treatment verification tools such as prompt gamma detector systems will be used to verify the depth of PT. Clinical implementation of these new technologies is expected to improve the accuracy and dose conformity of PT in the treatment of localized prostate cancers, and lead to better clinical outcomes. Improvement in dose conformity may also facilitate dose escalation, improving local control and implementation of hypofractionation treatment schemes to improve patient throughput and make PT more cost effective.

## 1. Introduction

Proton therapy (PT) was first proposed by Robert R Wilson in 1946 [1] because of the unique dosimetric property of proton beams known as the Bragg peak. In theory, a proton beam traversing a medium deposits a relatively small amount of radiation before it reaches the Bragg peak, and no radiation beyond it. The depth of the Bragg peak in the medium is determined by the energy of the protons. By irradiating the medium with proton beams of different energies, a range of spread-out Bragg peaks (SOBPs) can be positioned at a pre-defined depth of the medium, as determined by the range of the beam energies. PT takes advantage of this dosimetric property to deliver a conformal therapeutic dose of radiation to the patient by positioning the SOBP of a proton beam, of appropriate field size and range of beam energies, to precisely cover the tumor volume without damaging the normal surrounding tissue. Treatment planning studies have demonstrated superior dosimetric benefits of PT plans compared with plans for conventional external beam radiotherapy (EBRT), such as intensity-modulated radiotherapy (IMRT) and volumetric modulated RapidArc therapy (VMAT) [2,3].

Treatment of patients with PT began around 1954 [4], but progress in the development of this modality has been slow due to numerous technical hurdles. Challenges for PT pioneers included the immaturity of the PT accelerator and dose-delivery systems in handling complex treatments, lack of knowledge on dosimetric properties such as the relative biological effectiveness (RBE) of proton beams, and lack of interest and incentive in the manufacturing industry to invest in developments of PT equipment [5]. This situation changed when the US Food and Drug Administration approved the clinical use of PT in 1988 [6], and the world’s first hospital-based PT system was established, at Loma Linda University Medical Center in California, in 1990. Since then, the number of hospital-based PT facilities has increased exponentially, with 104 PT treatment facilities operating globally by the end of 2021 and many more under construction and planning [7]. By the end of 2020, over 250,000 patients had been treated with PT worldwide [7]. Although prostate cancer has been one of the main treatment sites in PT, the practice has been controversial because of a lack of clinical evidence to justify the treatment cost [8]. Reported clinical outcomes in both local control and toxicity of PT for prostate cancer are similar to those for EBRT. The theoretical dosimetric benefits of PT have not translated to greater clinical benefits than EBRT for the prostate, as they have in other diseases sites, including pediatric malignancies of the central nervous system, large liver cancers, ocular melanomas, chordomas and chondrosarcomas [9,10]. The far-below-expected performance of PT in treatment outcomes in prostate cancer has been attributed to two main causes. First, the results of the randomized controlled Androgen Suppression Combined with Elective Nodal and Dose Escalated Radiation Therapy (ASCENDE-RT) trial [11] point to inadequate dose prescription, particularly in patients with high-risk disease, indicative of a dose–response characteristic of radiotherapy (RT) of the prostate. Second, improper dose delivery due to large uncertainties associated with the individual processes involved in PT and pre-treatment workflow may compromise the dose conformity of PT treatment, as demonstrated by robust planning [12]. The objectives of this review are twofold: (1) examine the underlying reasons behind the disappointing clinical outcomes in PT treatment of prostate cancer; and (2) investigate new PT technologies and innovative work procedures that are being implemented, how they may improve the accuracy and quality of PT treatment and facilitate a reduction in treatment margins, and the possibility of dose escalation to the appropriate level.

## 2. Potential Benefits of PT and Reported Clinical Outcomes of Prostate Treatments

**Figure 1:**
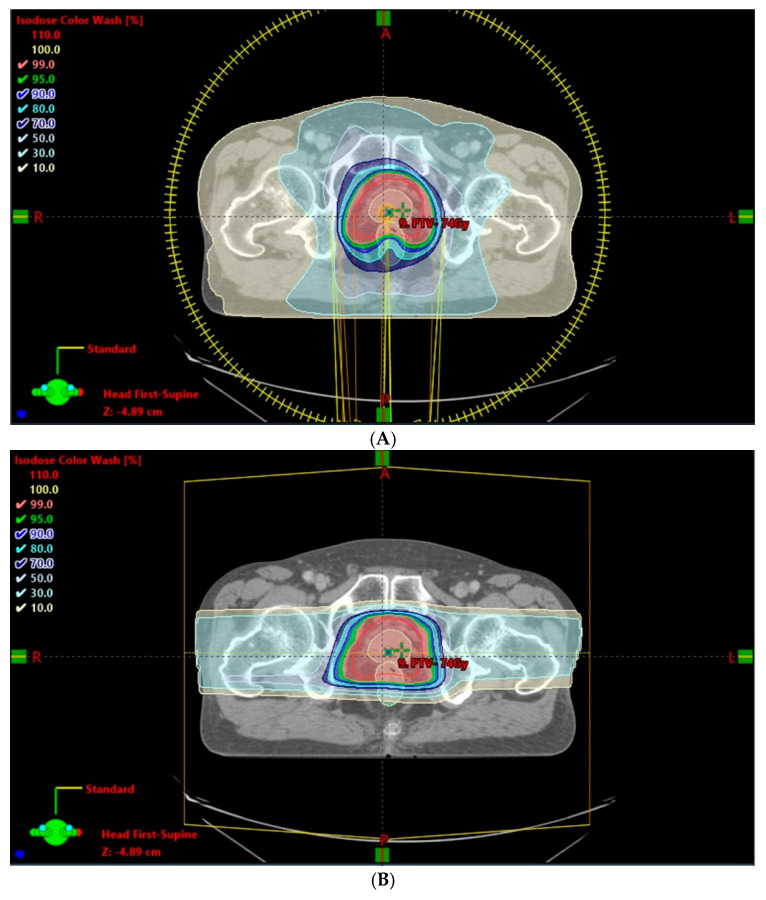
Integral dose comparison of volumetric modulated RapidArc therapy (VMAT)-full rotation (**A**) and photon therapy (PT)-bilateral (**B**) in prostate treatment plans. The most significant differences were in the 10% and 30% isodose distributions of the 78-Gy prescription.

Given the different beam characteristics of protons and photons, a modulated proton beam confines dose deposition to the target region, with a rapid depth- and lateral-dose fall-off favoring normal tissue sparing and target dose escalation. Several dose–volume comparison studies showed that both passive scattering [13,14] and pencil beam scanning [2,15] PT reduced the mean dose and percentage of volume receiving low dose in the rectum and bladder compared with IMRT. In the prostate, PT maintained dose coverage, demonstrating superior dose conformity over IMRT. In most circumstances, a simple bilateral proton beam is employed for localized prostate cancer. Other arrangements, such as anterior–posterior or anterior–oblique beams may be used in patients with a hip prosthesis or previously irradiated hip [16,17,18]. Nevertheless, PT has consistently been shown to have a smaller irradiated volume of normal tissue and lower integral non-target dose [14,15], as illustrated in Figure 1. A systematic review showed that the risk of radiation-induced second primary cancer appears to be small (range, 1 in 290 to 1 in 220 over all durations of follow-up), but does increase over time [19]. As prostate cancer survival improves, the risk of radiation-induced second malignancies becomes more relevant [20].

There is diverging evidence regarding the superiority of PT over IMRT in terms of disease control, survival and toxicity. A retrospective study at Loma Linda University reported overall 5- and 8-year actuarial biochemical disease-free survival rates of 75% and 73%, respectively, for PT in localized prostate cancer [21]. Another study compared the 5-year biochemical recurrence-free survival (bRFS) rates for intermediate-risk prostate cancer patients after surgical intervention and RT, which were 85.7% and 89.5% in patients treated with EBRT and permanent seed implant, respectively [22]. A randomized trial showed a similar biochemical failure rate for localized prostate cancer treated with high-dose PT and permanent brachytherapy [23]. Recently, a single-center retrospective study in Japan showed that 5- and 10-year freedom-from-biochemical-relapse rates were 93% and 86%, respectively, for intermediate-risk prostate cancer patients receiving 74 Gy (RBE) in 37 fractions of PT [24]. Apart from disease control and survival, several patient-reported prospective studies compared genitourinary (GU) and gastrointestinal (GI) toxicity, as well as sexual domain issues, between photon- and proton-based treatments [25,26,27]. Two studies, with follow-up duration up to 2 years, reported no differences in the GU, GI or sexual quality of life (QoL) scores [25,26]. Massachusetts General Hospital reported improvement in bowel QoL at 2–3 months after PT, but worsened urinary QoL at 1 year after PT. At the 2-year follow-up, however, there were no differences in either bowel/rectal QoL or urinary QoL between photon- and proton-based treatments [25]. Other claim-based studies showed degraded GI QoL [8,28,29]. Currently there are limited randomized trial data with which to evaluate the efficacy of proton- versus photon-based treatments, and various single-center/institute studies have reported mixed data. Therefore, a consensus has yet to be reached.

The theoretical dosimetric advantages of PT have not borne fruit clinically. Dose prescription might be a factor in the suboptimal clinical outcomes of PT. Numerous randomized trials have compared various dose prescriptions (range, 64 to 86.4 Gy) of 3D-conformal RT or IMRT, in which higher dose prescription demonstrated superior biochemical tumor control and decreased the risk of distant metastases for prostate cancer in different risk groups [30,31,32,33,34,35,36]. In the ASCENDE-RT trial, the low-dose rate brachytherapy (LDR-PB) boost arm received an iodine-125 implant prescribed at the minimum peripheral dose of 115 Gy, while the dose-escalation external beam (DE-EBRT) boost arm received an additional 32 Gy (total, 78 Gy) in 16 fractions. The LDR-PB boost patients were twice as likely to be free of biochemical failure at a median follow-up of 6.5 years [37]. Recalling the two largest single-center studies, a PT dose of 74 Gy, which lies in the middle of the dose-prescription spectrum, was employed. The authors speculated that there was room for escalating the dose prescription with PT, provided that the dose was delivered precisely and accurately.

Another factor limiting the potential of PT could be discrepancies between planned doses and actual doses delivered to patients. Major contributors to such discrepancies include uncertainties involved in the treatment planning and delivery processes, as well as technological limitations in PT treatment systems. Proton beams manifest an intrinsic range uncertainty due to range straggling, in which the absolute straggling width increases with energy. Water equivalent thickness is a one-dimensional range estimate in a heterogeneous medium. Monte Carlo (MC)-based studies have demonstrated that not only is the range related to water equivalent thickness, but it is also sensitive to the geometry and position of tissue density variation relative to the depth of the Bragg peak [38,39,40,41,42,43]. A study analyzed the inter-fraction motion of prostate cases with daily volumetric megavoltage X-ray computed tomography (CT). It showed that the higher uncertainties for inter-fraction range changes in lateral beams compared to anterior beams are likely due to the relative positions of the femoral head and the prostate between fractions of PT [44]. Range uncertainty can also be related to stopping power ratio (SPR) conversion. SPR is commonly converted from the Hounsfield unit (HU) in CT images using a generic look-up table. Studies have analyzed the uncertainties from various sources, including CT image noise [45], artifacts [46,47], tissue composition variation [48,49,50,51], and others. A notable study by Yang et al. reported that the 95th percentile of composite range uncertainty in the prostate region is 3% [48]. Apart from range uncertainty, setup error and inter-/intra-fraction organ motion also played important roles in dose delivery accuracy because of the steep dose gradient in PT. A study by Park et al. [52] compared dose variations upon the introduction of various systematic and random setup errors (Gaussian distribution with standard deviation of 2 mm) in proton clinical plans for prostate cancer. Although the target coverage in the prostate was retained (−0.3% to nominal plan), the standard deviation of V_45Gy_ for the rectum was 5.6%, suggesting that noticeable deviations from the nominal plan may result from setup errors [52].

The RBE of PT has typically been taken as a spatially invariant value of 1.1, regardless of clinical endpoint [38]. However, dose- and depth-dependence of RBE has been demonstrated in which the RBE values in the distal edge rose to 1.21 [53,54]. A wide RBE range was reported in low α/β biological systems, with RBE values often above 1.1 within the SOBP linear energy transfer (LET) range, compared with the lower RBE values of high α/β systems (below 1.1). Dose per fraction was also reported to have an impact on RBE for low α/β biological systems [55]. Based on existing in vitro and in vivo studies, the RBE uncertainty in normal tissue is higher (compared to the nominal value of 1.1), potentially causing unexpected complications and toxicity.

## 3. Opportunities for Improvement with New Technologies and Innovative Techniques

**Figure 2:**
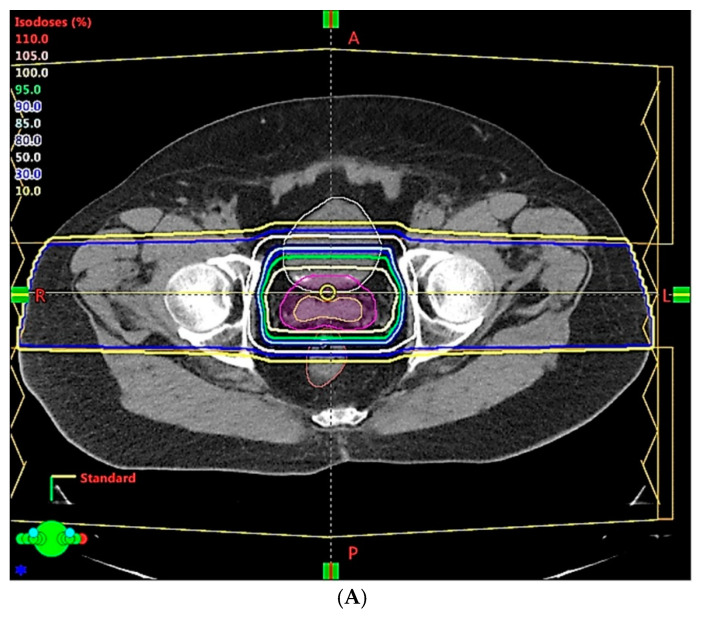

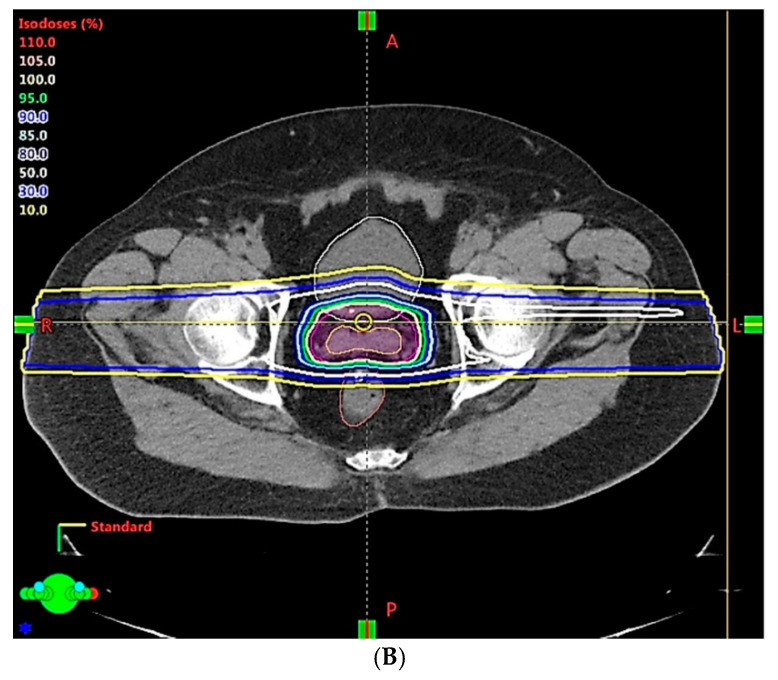
Isodose distribution comparison of two different proton therapy techniques: conventional passive scattering (**A**) and modern intensity-modulated proton therapy (IMPT) (**B**). IMPT shows improved dose conformity near critical organs.

For decades, PT treatment systems with double scattering or a wobbling beam with brass aperture collimation and compensator have been used to treat prostate cancer. Large treatment margins of up to 10 mm or more were used in “large field” conventional PT techniques [50,56], and contributed to critical organ toxicity [57]. Most of the reported clinical data were based on treatments delivered with this large field technique. With the recent commercial availability of the latest generation of PT technologies, clinical implementation of new techniques such as intensity modulated proton therapy (IMPT) can now be realized. IMPT is capable of delivering treatment with superior dose conformity, which can potentially provide better protection of critical organs, as illustrated by the dose displays of two treatment plans shown in Figure 2. In principle, a reduction in the regions of critical organs that are exposed to high radiation doses will help reduce related toxicity [15]. In reality, dose conformity of IMPT treatment plans may not always be reproducible in a patient’s course of treatment because of the various uncertainties involved in treatment and pre-treatment processes.

Proton range uncertainty is one factor that can severely compromise dose conformity and affect treatment outcome. For instance, in their study on head and neck treatments, Tattenberg et al. found that a reduction from the currently adopted range uncertainty of approximately 4% to a potentially achievable level of 1% could reduce the probability of brainstem necrosis by up to 1.3% in the nominal scenario, and by up to 2.9% in the worst-case scenario [58].

Proton range uncertainties are mainly caused by uncertainties arising from: (1) errors introduced when deriving the mass density or SPR for PT dose calculation in treatment planning; and (2) patient positioning errors introduced during treatment setup. We have performed simulated studies on the dosimetric effects of different robust optimization scenarios of range and setup uncertainties in IMPT prostate plans. The results from nominal plans are presented in Table 1, and clearly demonstrate a dependence of critical normal organ dose on setup uncertainties. There is a minor dosimetric effect for range uncertainty because of the bilateral delivered beam in prostate treatment. The non-target tissue was defined as body tissue that excludes the clinical target volume (CTV) and limits the region between 1 cm superior and inferior from the CTV. Such uncertainties should be minimized before the dosimetric benefits of PT can be realized as clinical benefits in prostate treatment.

In traditional PT of prostate cancer, large margins are used to account for the two major sources of uncertainties mentioned above. Such planning strategies may compromise the dose conformity of the treatment, thereby affecting the clinical outcome. These uncertainties can now be minimized using new technologies and innovative techniques in the PT workflow, as discussed below.

1. The large uncertainties in CT HU values, and CT conversion to mass density or SPR [45,50], can now be overcome by modern CT, which can acquire mass density maps or SPR directly [59] from CT image reconstruction data. Mass density and SPR are less sensitive to patient scan conditions than HU [60], and thus have less uncertainty. With this approach, the range uncertainty could be decreased from 3.5% to 2–2.5%. There is also an increasing interest in dual energy CT (DECT) as an alternative imaging modality for PT treatment planning because of its ability to discriminate between changes in patient density and chemical composition [61]. SPR calculation accuracy was found to be superior, on average, for DECT relative to single energy CT (SECT). Maximum errors of 12.8% and 2.2% were found in SPR data derived from SECT imaging and DECT imaging, respectively [61]. Quantitatively, the maximum dose calculation error in the SECT plan was 7.8%, compared to a value of 1.4% in the DECT plan [62]. Additionally, a novel spectral CT imaging technique based on a dual-layer detector-based approach has been used to demonstrate improvement in SPR prediction for particle therapy treatment planning, and would minimize the beam range uncertainty, allowing for the use of reduced safety margins in patient plans [63].
2. Because of the inferior soft tissue contrast, orthogonal X-ray imaging systems rely on bony structures for verification of treatment position during patient setup. This type of setup technique can result in large positioning errors due to daily movement of the target and organs at risk (OARs) relative to the bony structures in the former technique. With fiducial markers implanted inside the prostate, many studies concluded that image registration by fiducial markers would reduce matching error. However, some patients may not accept marker implantation. Migration of markers with time may introduce registration errors. Such problems can now be minimized using on-board cone beam CT (CBCT). The better image quality of CBCT can provide 3D images and more information on the anatomic relationships between organs [64,65], which can be used to improve the accuracy of patient setup. Besides patient positioning, CBCT images can also provide information about inter-fractional changes in patient anatomy. In a recent study, an image-based correction method to generate pseudo-CT images from CBCT images was investigated for possible application in proton dose calculations [66] in adaptive PT. MRI, which has the ability to offer fast real-time imaging with high soft tissue contrast in the absence of ionizing radiation exposure [67], is being investigated for use in patient setup in RT. Our study using an external MRI setup room [68] and studies by others [69] indicated that patient positioning accuracy on the order of 1 mm is feasible, and is a significant reduction from that of conventional setup systems.

**Figure 3:**
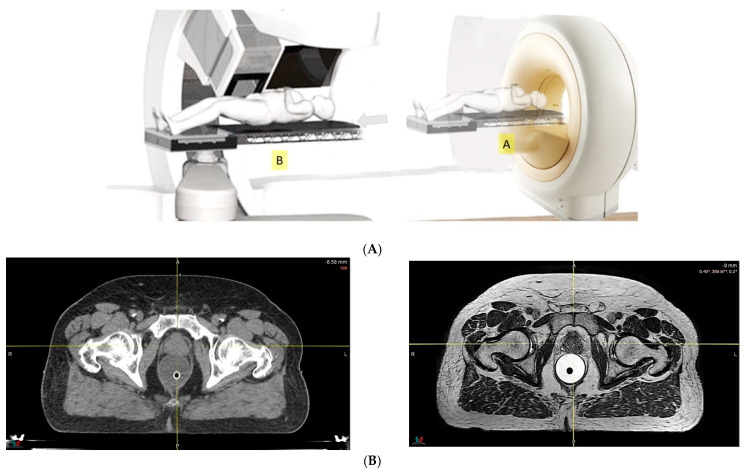
(**A**) Diagram of a proton therapy system with in-room magnetic resonance (MR). MR images are acquired first, with the patient lying on a robotic couch in position A, followed by relocation to position B for proton delivery, directly after image registration. (**B**) Comparison of computed tomography and MR images of prostate cancer.

Improving the levels of accuracy in determining the penetration ranges of proton beams and positioning patients for treatment can facilitate better organ sparing, as illustrated in Table 1. Reduction in PT range and patient positioning uncertainties can also help improve dose conformity in PT treatment, leading to better protection of normal tissues and reduced toxicity, and facilitating the possibility of dose escalation to improve local control. The concept of in-room or integrated MRI-guided PT, although still investigational, is gaining momentum [70,71,72]. Integrated PT with MR gantry systems have created many engineering problems that must be overcome. Deflection of complex charge particles observed in magnetic fields changed the direction vectors, depending on the energy and heterogeneity. Optimized IMPT plans should account for the scanning beam in the complex fringe field. Compared with integrated MRI-guided PT, there is less uncertainty in a PT system combined with in-room MRI equipment. With in-room or on-line MRI-guided patient positioning, as illustrated in Figure 3 (upper panel), further improvements in accuracy and dose conformity in PT treatment are possible, and we should remember that MRI delivers superior soft-tissue contrast compared with CT (lower panel). Development of such a treatment modality will be technologically and economically challenging, and is unlikely to be accomplished in the near future.

Significant advancements in treatment planning system (TPS) technology have also been made in recent years. The MC-based TPS dose calculation algorithm is considered to be the most accurate method, and it has been demonstrated that it could lead to a significant reduction in treatment planning margins [50,73]. However, the lengthy time required for MC-based dose calculations in the past was impractical for routine clinical treatment planning. In recent years, computer graphic processing units (GPUs) have drawn great attention due to their tremendous ability to accelerate a variety of computationally intensive tasks. A TPS running on a GPU computer makes the MC-based dose calculation algorithm in RT treatment planning feasible. TPS options based on simplified MC algorithms and GPU computers are now commercially available for routine clinical use. Because of these efforts, the calculation time of MC-based proton dose calculation has been significantly shortened. MC-based dose planning and calculation in PT is now becoming routine, helping to reduce treatment uncertainties. Additionally, the resolution of dose grid and MC simulation uncertainties are improved with a GPU-based TPS, further improving the dosimetric accuracy of the treatment plans.

As mentioned earlier, PT is very sensitive to changes in the position and shape of the target and OARs in patients, both between treatment fractions and within a fraction. With reduction in treatment uncertainties and higher dose conformity, accurate quality assurance (QA) verification of the treatment plan, especially treatment geometry at every treatment fraction, is essential. Several efforts [74] are ongoing to develop new QA tools and techniques that can be used to ensure geometric and dosimetric accuracies of PT treatments. QA tools and techniques based on proton radiography and tomography, positron emission tomography, and prompt gamma imaging are under investigation. Currently, verification of proton range in vivo is mostly based on detection of secondary radiation, such as prompt gamma rays [75]. Various approaches to on-line monitoring of PT have been reported, and some have already been successfully tested clinically with patients [76]. A recent European Society for Radiotherapy and Oncology conference (ESTRO 2020) revealed a new study of on-line MRI that verified the proton beam range of PT [77]. PT range verification using positron emission tomography has been investigated at several institutions [78].

TPS with robust planning optimization that accounts for uncertainties that cannot be corrected (e.g., movement of the target and critical OARs during treatment) are in routine clinical application. This technique helps to predict and illustrate the actual dose to be delivered to the patient, which in turn can further improve dose optimization and clinical decision making. The uncertainty in RBE is a dosimetric issue under active investigation, with the aim to improve the accuracy of patient dose. TPS dosimetry algorithms are available that test the dependency of RBE on the LET of protons. It is well-known that the current adopted average RBE value of 1.1 assumes that proton doses are about 10% more biologically damaging than MV X-ray RT. However, experimental data indicate that RBE depends on dose, LET and α/β ratio [79,80,81,82,83], as protons traversing a patient change energy as they travel. They have higher LET at the distal end of an SOBP, and such variations, if not taken into account in dose calculation, can theoretically have an impact on patient dosimetry. Clinical evidence on the effect of RBE variations on treatment outcome has yet to be reported. By contrast, biological treatment planning and LET-guided dose optimization have been developed and commercially available for testing and clinic planning studies. They are mainly used in generating reference plans with LET-based dose optimization for guiding the planning of complex treatment involving critical normal tissue structures [84,85,86]. Subject to satisfactory demonstration of clinical evidence, LET-based dose optimization may potentially improve the quality of PT treatment plans.

MC-based algorithms calculate the variable RBE from the LET of the proton interactions at different locations in the patient, and incorporate the variable RBE data into patient dose calculation and optimization. As a result, a theoretically more accurate dose map of the target and OARs of the treatment can be constructed. This information can serve as a valuable reference for protecting OARs from excessive dose [87,88]. In the case of prostate treatment, if variance of RBE is incorporated into robust optimization, biological dose for critical organs such as the rectum and bladder could be minimized to protect these organs.

Additionally, LET-based dose optimization will be applied to the treatment plan, which provides a useful reference map for plan assessment. All of the new technologies mentioned above provide us with the appropriate tools to go a step further with treatment of the prostate using PT. DECT and spectral CT reduce conventional CT uncertainty and handle well-defined shrunken tumor margins. By applying the use of implanted fiducial markers, CBCT or in-room MR imaging guided system, setup errors could be controlled to within a relatively small amount. Furthermore, the new technological developments described above will better protect critical organs from unnecessary radiation dose.

## 4. Potential Clinical Benefits and Cost Effectiveness of PT in Treatment of Prostate Cancer

The overall survival of localized prostate cancer patients treated with PT, photon-based EBRT, and brachytherapy in the US between 2004 and 2015 were analyzed and compared by Liu et al. [89]. They concluded that PT was associated with a significant overall survival benefit compared to EBRT, and delivered outcomes were similar to brachytherapy. PARTIQoL, an ongoing phase III randomized trial [NCT01617161], will determine whether IMRT or PT best minimizes the side effects of treatment for low- or intermediate-risk prostate cancer. Another ongoing study, COMPARE [NCT03561220], will compare the patient-centric outcomes (quality of life, toxicity, and disease control) between parallel cohorts of men with prostate cancer treated simultaneously at PT facilities and at geographically similar conventional IMRT facilities. With the latest generation of PT technologies and high-precision innovative treatment and pre-treatment techniques that have been developed to improve the accuracy and quality of PT treatment, we can soon expect to see clinical data with more favorable outcomes in prostate treatment using PT. Dose escalation is one treatment strategy that may now be safely implemented to improve local control, and yet an important question remains: is dose escalation with PT superior to photon-based treatment? Based on the fundamental principles of dose–response characteristics in RT, it is likely that the dosimetric advantages of PT, particularly regarding dose escalation, will translate into clinical benefits and outperform conventional EBRT. With better dose conformity, more appropriately escalated dose prescriptions to the target, together with clinically acceptable OAR dose constraints, can be used in PT treatments. Additionally, the dosimetric advantages of PT in prostate treatment can be enhanced further by the use of rectal spacers [90,91], particularly when IMPT is used [92,93]. Apart from toxicity, studies comparing IMRT versus 3D-conformal RT [94] and IMRT versus PT [95] suggest that conformity of the treatment is associated with the risk of radiation-induced second malignancies. Based on organ equivalent dose calculations, the risk of secondary malignancies in out-of-field organs, including the stomach, lungs and thyroid, was at least 5 times higher for IMRT than for PT in prostate treatment. In the prostate, smaller irradiated volume to normal tissue and lower integral non-target dose in PT may reduce the risk of secondary malignancies. Furthermore, a lower OAR dose from previous delivery also favors re-treatment upon recurrence and successive treatment [96].

The cost-effectiveness of PT has been questionable, mainly due to the high infrastructure and machinery costs [97]. In a systematic review that analyzed the costs and cost-effectiveness of PT for different disease types [98], Verma et al. indicated that the cost-effectiveness of PT is generally lower for low-risk prostate cancer. While patient selection plays a crucial role in such analyses, with considerations of treatment efficacy and costs associated with acute/late toxicities, we note that the cost of PT is coming down as the technology advances. The weight and size of accelerators and gantries have been decreased by half since the 1990s [99], and novel designs with superconducting magnets have been proposed that would significantly scale down their sizes even further [100]. The upfront costs for infrastructure and machine units are on a downward trend. Regarding operational costs, IMPT is theoretically cost saving compared with passive scattering PT, which involves the use of a patient-specific brass aperture and compensator; however, few studies have discussed this cost implication [9]. Moreover, a number of clinical trials have suggested a higher therapeutic ratio for hypo-fractionation in low α/β prostate cancer [101,102,103]. Improvement in dose conformity in PT treatment may allow hypo-fractionation treatment schemes to be implemented as the standard of care, which can effectively increase patient throughput [104]. The concept of remote patient positioning in the patient setup room may also increase patient throughput [105,106]. Collectively, these efforts at making PT more accurate and efficient will surely improve the efficacy and cost effectiveness of PT in the treatment of prostate cancer.

## 5. Conclusions

Despite its dosimetric advantage, the use of PT for the treatment of prostate cancer has been limited, mainly by inadequate dose prescription and improper dose delivery. Advanced high-precision PT technologies, dose planning and beam delivery techniques have been developed and commercially available for implementation and are expected to optimize the dose conformity of PT in prostate treatment. Improvement in dose conformity can enhance local disease control, patient throughput and cost-effectiveness by allowing for implementation of dose escalation and hypofractionation treatment schemes.

## Figures and Tables

**Table 1:** Comparative effects of simulated robust optimization uncertainties in proton beam range and setup on dosimetry in a prostate intensity-modulated proton therapy (IMPT) planning system.

Clinical Scenarios									
Range uncertainty	3.5%				3.0%	2.5%	2.0%	1.5%	1.0%
Setup error	5 mm	3 mm	2 mm	1 mm	1 mm				
CTV, V_78Gy_	99.9%	99.6%	99.1%	98.4%	98.8%	98.5%	98.8%	99.3%	99.6%
Rectum, V_70Gy_	30.8%	25.9%	24.5%	20.1%	19.7%	20.1%	18.2%	19.5%	19.5%
Bladder, V_70Gy_	35.2%	30.6%	29.1%	25.3%	24.7%	24.7%	24.5%	23.6%	23.5%
Rectum, D_mean_ (Gy)	37.1	33.3	32.4	28.8	28.5	28.9	27.1	28.4	28.5
Bladder, D_mean_ (Gy)	40.4	37.0	36.1	32.8	32.3	32.5	32.2	31.8	31.7
Non-target tissue, D_mean_ (Gy)	14.6	13.6	13.3	12.3	12.1	12.1	11.7	11.8	11.7

Non-target tissue describes body tissue that excludes the clinical target volume (CTV) and limits the region between 1 cm superior and inferior from the CTV.
